# Comparison of the Effects of Music Therapy and Aromatherapy on Physiological Indices in Surgical Patients: A Randomized Parallel-Group Trial

**DOI:** 10.1155/sci5/6187659

**Published:** 2025-11-26

**Authors:** Ziba Bolandi, Alireza Khatony, Mansour Rezaei, Mahbobeh Sajadi

**Affiliations:** ^1^Nursing Department, Arak School of Nursing and Midwifery, Arak University of Medical Sciences, Arak, Iran; ^2^Infectious Diseases Research Centre, Health Policy and Promotion Institute, Kermanshah University of Medical Sciences, Kermanshah, Iran; ^3^Social Development and Health Promotion Research Centre, Kermanshah University of Medical Sciences, Kermanshah, Iran

**Keywords:** aromatherapy, intensive care units, music therapy, rosmarinus, vital signs

## Abstract

Maintaining stable physiological indices is essential in postoperative ICU care, yet the effects of music therapy and aromatherapy on these indices remain limited and inconclusive. This study compared the effects of music therapy and 25% rosemary aromatherapy on ICU patients recovering from surgery. Ninety-two general surgery patients were randomly assigned to four groups: music therapy, aromatherapy, combined therapy (music and aromatherapy), or control. Data included demographic information and physiological indices. Music therapy involved 30 min of instrumental music; the aromatherapy group received 25% rosemary oil; the combination therapy group received both; the control group received a placebo aroma and turned off the headphones. Analysis with SPSS-18 showed that combination therapy and aromatherapy significantly reduced respiratory rate. Only the combination therapy group showed a notable increase in oxygen saturation. Aromatherapy also significantly decreased pulse rate, while neither music therapy nor control had significant effects on respiratory rate. No group showed significant changes in blood pressure. These noninvasive, cost-effective interventions are recommended for ICU care.

**Trial Registration:**

Iranian Registry of Clinical Trials (IRCT): IRCT20100913004736N27

## 1. Introduction

Patients undergoing surgery and admitted to intensive care units (ICUs) face a variety of environmental challenges beyond the procedures themselves [1]. Factors such as isolation, restricted mobility, and noise pollution from monitoring devices [2] can have detrimental effects, leading to sleep disturbances, pain, stress, anxiety, and alterations in physiological indicators, including blood pressure, heart rate, respiratory rate, body temperature, and arterial blood oxygen saturation (SpO_2_) [3]. Monitoring these indicators is essential for healthcare providers to assess patients' clinical status accurately [4]. Stabilizing and improving these physiological indices is a primary therapeutic goal, often pursued through both pharmacological and nonpharmacological approaches [5, 6]. However, while pharmacological interventions are effective, they are often accompanied by side effects such as nausea, vomiting, drowsiness, and allergic reactions [7].

As a result, complementary medicine, particularly music therapy and aromatherapy, has emerged as a promising alternative for stabilizing and enhancing physiological indices [8]. These approaches are appealing due to their accessibility, affordability, and minimal side effects when compared to pharmacological treatments [9]. In the noisy environment of ICUs, music-based interventions can act as effective distractors, helping to reduce pain, alleviate anxiety, and modulate physiological indicators [10]. Research has shown that music-based interventions can positively impact physiological indices [5, 11, 12]; however, some studies have reported no significant effects on parameters such as blood pressure, heart rate, or respiratory rate [13, 14].

Aromatherapy, another nonpharmacological intervention, has also shown potential in stabilizing and improving physiological indicators [15]. This therapy employs plant essences or extracts, such as lavender, rose, orange blossom, sandalwood, and rosemary [4], administered through massage, inhalation, bathing, or topical application to enhance mental and physical well-being [16]. Incorporating patient-preferred essential oils appears to enhance treatment outcomes [17]. Aromatherapy works by stimulating the limbic system and hypothalamus, reducing corticotropin hormone release and thereby lowering cortisol levels. This, in turn, leads to muscle relaxation, improved circulation, and better respiratory function [4, 18].

Rosemary (*Salvia rosmarinus)*, a member of the Lamiaceae family, contains beneficial compounds such as caffeic acid, carnosic acid, chlorogenic acid, and rosmarinic acid [19, 20]. Classified as “generally safe” by the Food and Drug Administration (FDA) [21], rosemary essential oil has been shown to promote the release of calming neurotransmitters such as enkephalins and endorphins, which may help reduce anxiety and stress [22]. In addition, rosemary oil's aroma has been found to stimulate the release of serotonin and dopamine in the brain, influencing emotional states [19]. Inhalation aromatherapy with rosemary essential oil has demonstrated effectiveness in modulating patients' physical conditions and may reduce anxiety and stress over the long term [23]. A review also supports rosemary's potential in addressing neurological disorders [24].

The existing literature presents mixed findings regarding the effects of music on physiological indicators. For instance, a systematic review and meta-analysis observed a trend toward reduced blood pressure in hypertensive patients following music interventions, although it did not establish a cause–effect relationship due to the lack of formal comparisons with control groups [13]. In contrast, other studies have reported significant improvements in systolic and diastolic blood pressure, heart rate, respiratory rate, SpO_2_, and body temperature [11, 12, 25]. Similarly, aromatherapy has been associated with reductions in heart rate, blood pressure, and oxygen saturation [26], while another study reported no significant effects on these parameters [27]. Interestingly, in a 2025 randomized clinical trial among endodontic patients, neither music therapy nor aromatherapy had a measurable impact on heart rate, as reductions observed were not statistically significant compared to the control group [28]. However, music-based interventions were found to be more effective than aromatherapy in lowering systolic blood pressure in hypertensive patients [29].

While rosemary contains several beneficial compounds and is considered safe by the FDA, research specifically examining its impact on physiological indicators is limited. This study seeks to fill this gap by directly comparing the effects of music-based interventions and rosemary aromatherapy on the physiological indicators of postsurgical ICU patients. By addressing the limitations of previous research and investigating the underlying mechanisms, this study aims to contribute to the development of effective and safe preventive and therapeutic interventions for ICU patient care. The findings from this study may also guide nurses in utilizing these nonpharmacological interventions as adjunctive treatments, thereby improving the quality of nursing care and patient outcomes.

The theoretical framework underpinning this study posits that music-based interventions can reduce stress and anxiety, subsequently leading to improved hemodynamic indices. Exposure to music activates specific brain regions, triggering responses that increase dopamine levels, potentially inducing feelings of pleasure and well-being [30, 31]. By fostering relaxation and modulating brain activity, music contributes to the regulation of stress hormones, thereby alleviating pressure on both the cardiovascular and respiratory systems. This, in turn, results in improved hemodynamic outcomes [32]. Several studies have demonstrated that music-based interventions effectively reduce stress and anxiety [33, 34], leading to favorable effects on hemodynamic parameters in hospitalized patients [11]. Building on this premise, the current study aims to compare the effects of music-based interventions and aromatherapy on the hemodynamic indices of ICU patients. The research questions guiding this investigation are as follows:

1. How does music therapy influence physiological parameters, including systolic and diastolic blood pressure, heart rate, respiratory rate, and SpO_2_, in postoperative patients?
2. What is the effect of inhalation aromatherapy with 25% rosemary essential oil on these physiological parameters in postoperative patients?
3. How does the combination of a music therapy and aromatherapy using 25% rosemary essential oil affect the same physiological parameters?
4. Which therapeutic approach, music-based intervention, aromatherapy, or their combination, exerts the greatest impact on improving and stabilizing physiological indicators in postoperative patients?

## 2. Materials and Methods

### 2.1. Study Design

This study, conducted from June 17, 2022, to September 18, 2022, employed a factorial design to simultaneously examine the effects of aromatherapy and music-based intervention. This rigorous methodology allows for the investigation of interaction effects between two independent variables, providing a comprehensive understanding of their interplay [35, 36]. This study's factorial design compared the combined intervention of aromatherapy and music therapy against each intervention alone. By examining interaction effects, this approach provides an evidence-based foundation for exploring the potential benefits of integrating these interventions to enhance patient care.

To ensure rigorous reporting standards, a parallel-group, randomized controlled trial design with a 1:1:1:1 allocation ratio was utilized. This research adhered to the Consolidated Standards of Reporting Trials (CONSORT) guidelines (Supporting file) [35].

### 2.2. Sample and the Sampling Method

This study was conducted at two hospitals in Kermanshah, Iran: Taleghani Hospital, a trauma center with two active ICUs, and Imam Reza Hospital, a large specialized center with five ICUs. The sample size was calculated to detect a clinically significant difference in anxiety scores between intervention and control groups. The effect size used for the sample size calculation was derived from prior research, specifically from the study by Goli et al., which reported mean anxiety scores of 31.2 ± 7.4 for the aromatherapy group and 39.7 ± 8.4 for the music therapy group [9]. Based on this, an effect size of 8.5 was estimated. Using a two-sided *t*-test with a significance level of 0.05 (Type I error = 0.05) and a power of 0.90 (Type II error = 0.10) and a pooled estimated SD of 7.9, a minimum sample size of 19 participants per group was determined. This calculation also accounted for a potential dropout rate of 20%, yielding 23 participants per group, with a total of 92 participants considered for this study.

#### 2.2.1. Participant Selection (Sampling)

Participants were selected using a convenience sampling method, whereby 92 participants were recruited from the postoperative wards of the two hospitals. Eligible participants were adults (18–65 years old) who underwent laparotomy or thoracotomy surgery and met the following inclusion criteria: willingness to participate in the study, full alertness (Glasgow Coma Scale [GCS] score of 15), intact hearing and olfactory senses, and no self-reported history of drug addiction, psychiatric disorders, thyroid diseases (due to their association with hemodynamic instability [37]), or allergies.

Hearing was assessed by the researcher whispering words and numbers from 30 cm behind the patient, who repeated the words and numbers to demonstrate normal hearing. Olfactory function was assessed by having patients close their eyes and identify the scent of cardamom placed near their faces.

Potential participants were excluded if they (1) declined to participate or continue participation, (2) experienced allergic reactions to the aromatherapy essence (e.g., redness, itching, cough, and sneezing), (3) were transferred to another ward, (4) experienced a change in their level of consciousness, or (5) died during the study period.

The first author screened potential participants, assessed their eligibility, and recruited them for the study. Sampling was completed based on reaching the target number of participants, with no dropouts occurring.

#### 2.2.2. Randomization

Following participant selection, a permuted block randomization technique was used to assign participants to one of the four study groups: Music therapy (A), aromatherapy (B), combined music and aromatherapy (C), and control (D). Using Sealed Envelope's web-based randomization service (https://www.sealedenvelope.com), 12 blocks of eight participants each were generated. The blocks followed an octet sequence (AABBCCDD and ABACCDBD) to ensure balanced allocation across groups.

To enhance allocation concealment, a double-blinded physical randomization procedure was implemented alongside the online service. Eight cards, each representing one study group, were placed in separate opaque envelopes, which were then placed in a box. The researcher responsible for enrollment and study conduct remained blinded to group assignments. Upon participant enrollment, a card was randomly selected from the box, revealing the assigned group. This process continued until all participants were allocated.

### 2.3. Study Instruments

Data collection involved a demographic information form and a physiological indicators' checklist. The demographic form captured details on age, gender, education, occupation, type of surgery, and the use of analgesic or anxiolytic medications. The physiological indicators checklist included monitoring of systolic blood pressure, diastolic blood pressure, heart rate, respiratory rate, and SpO_2_, all measured using a digital monitoring system.

The ARAD P10 model (Saa-Iran, Iran) was the system employed for monitoring, which included a display, blood pressure cuff, temperature sensor, and pulse oximeter. Annual inspection and calibration of these systems by Saa-Iran Company experts ensured their accuracy and reliability. For the music-based intervention, a Lenovo music player (Lenovo, China) and Lito headphones (Lito, China) were used.

### 2.4. Interventions

The researcher conducted daily visits to the ICUs to identify and enroll eligible patients. After confirming full consciousness, the researcher provided a detailed explanation of the intervention process and obtained informed consent from participants before enrollment. Baseline data collection included completing a demographic information form and recording physiological indicators such as blood pressure, heart rate, respiratory rate, and SpO_2_.

To measure blood pressure, a standardized procedure was followed. A 15 cm-wide blood pressure cuff was fully deflated and securely attached to the patient's right arm. The researcher then initiated the measurement by pressing the start button on the monitor's blood pressure cuff pump, recording the systolic and diastolic blood pressure from the monitor's display.

SpO_2_ assessment was performed using the patient's left index finger. After ensuring the absence of nail polish and that the finger was warm, the pulse oximeter probe site was cleaned with alcohol and securely attached. Once the monitor display stabilized, the SpO_2_ level was recorded.

**Figure 1:**
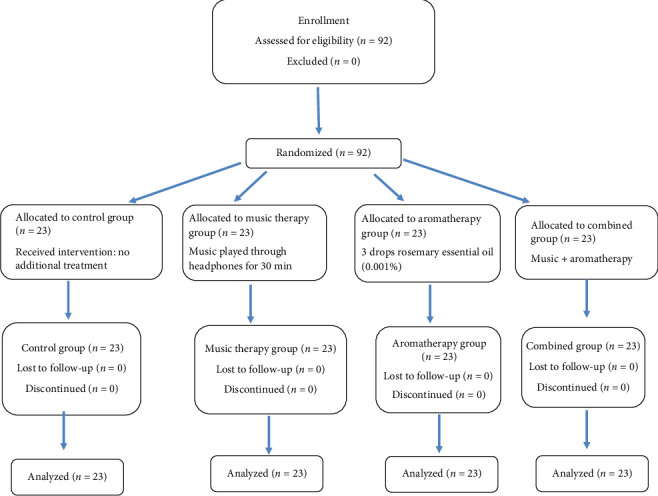
The CONSORT diagram of the study.

For temperature measurement, the digital thermometer sensor was disinfected with alcohol and placed in the patient's armpit. After the display stabilized, the temperature was recorded on the designated measurement form. Following the baseline physiological measurements, participants were randomized into one of the four groups: music-based intervention, aromatherapy, combined intervention, or control.  Figure 1 visually depicts the study process.

#### 2.4.1. Music-Based Intervention

Participants received a 30-min session of instrumental traditional music via headphones. Patients were allowed to choose their preferred music from six culturally relevant instrumental pieces: “Bar Samā” by Sohrab Pournazeri, “Beydād” by Parviz Meshkātiān, “Kurd Bayāt” by Jalāl Zolfonun, “Isfahan” by Farhang Sharif, “Jam-e Dorān” by Keyvān Sākht, and “Sāye Roshan” by Keyhān Kalhor. These slow-tempo pieces, selected based on recommendations from music experts, aimed to induce relaxation and calmness.

Environmental sounds were minimized during the sessions to create an optimal therapeutic atmosphere. The timing of the music sessions was adjusted carefully to avoid interference with other treatments. Volume levels were customized to each patient's preference to ensure comfort. Patients were instructed to lie comfortably in bed throughout the session to enhance relaxation and optimize the therapeutic impact of the music.

#### 2.4.2. Aromatherapy

Rosemary essential oil (25% concentration), produced by Tabibdarou Pharmaceutical Company (Kashan, Iran), was utilized. This concentration was prepared by mixing 25 mL of 100% rosemary essential oil with 75 mL of odorless liquid paraffin. During the intervention, three drops of rosemary essential oil were applied to a cotton ball and affixed to the patient's collar. Patients were instructed to inhale gently for 30 min. The essential oil was stored in opaque containers at 2°C–8°C to preserve its quality.

#### 2.4.3. Combined Intervention

Participants received both the music-based and aromatherapy interventions concurrently, as described above.

#### 2.4.4. Control Group

To control for potential effects of headphone use and olfactory exposure, participants in the control group wore headphones with the sound turned off and were exposed to a placebo aroma pad. The placebo consisted of 0.5 mL of the 25% rosemary essential oil diluted in 100 mL of odorless liquid paraffin. Three drops of this solution were applied to a cotton ball, and patients were instructed to breathe calmly and naturally for 30 min.

#### 2.4.5. Outcome Assessment and Blinding

A trained outcome assessor, blinded to group allocation, conducted all physiological measurements both before and after the interventions. The assessor remained fully unaware of the group assignments throughout the study and had no involvement in delivering the interventions. To ensure blinding, the assessor was kept physically separate from the intervention sites and was only provided with coded participant IDs, with no reference to group assignments.

For data analysis, all data were coded, and group assignments were concealed until the analysis was fully complete. Communication between the research team responsible for intervention delivery and the outcome assessor was strictly limited to logistical issues, ensuring no discussions related to the interventions or group allocation occurred.

#### 2.4.6. Standardized Measurement Protocol for Physiological Parameters

To evaluate the interventions' impact on physiological health indicators, temperature, SpO_2_, heart rate, and respiratory rate were measured before and immediately after each intervention. Considering the short interval (approximately 30 min) between pre-and postintervention measurements, the potential for significant natural variability in physiological parameters over time was minimized. However, to further reduce the possibility of natural fluctuations, we employed several methodological safeguards.

1. Timing of measurements: All measurements were consistently taken immediately postintervention, mitigating diurnal variations and reducing the influence of external factors on the readings.
2. Patient positioning: Throughout the study, participants were kept in a consistent semi-Fowler's position during all measurements, ensuring that positional effects on physiological parameters were minimized.
3. Equipment calibration: All measurement devices were calibrated according to the manufacturer's specifications before each measurement session to ensure accuracy and consistency.

### 2.5. Methods of Potential Bias Control

To mitigate potential biases, the following strategies were employed: (1) Randomization of participants reduced selection bias, ensuring equal distribution of potential confounders across groups [38]. (2) Blinding the study statistician to group assignments minimized detection bias. (3) Attrition bias was addressed by actively engaging participants and communicating study expectations. (4) Statistical tests performed before the study confirmed the homogeneity of the study groups, indicating comparability across variables.

### 2.6. Statistical Analysis

Data analysis was conducted using IBM SPSS Statistics, Version 16 (https://www.ibm.com/products/spss-statistics), employing both descriptive and inferential statistics. Descriptive statistics included mean, standard deviation, frequency distributions, and percentages. Inferential statistics involved the Kolmogorov–Smirnov test, analysis of variance (ANOVA), Tukey's post hoc test, Kruskal–Wallis test, chi-square test, paired *t*-test, and Wilcoxon test. The Kolmogorov–Smirnov test was applied to assess data normality. Age and pre- and postintervention diastolic blood pressure were normally distributed, whereas pre- and postintervention systolic blood pressure, heart rate, respiratory rate, and SpO_2_ were not.

The chi-square and ANOVA tests were used to evaluate group homogeneity for qualitative variables (gender, education, marital status, surgical history, type of surgery, and analgesic use) and normally distributed quantitative variables (age and pre- and postintervention diastolic blood pressure). The Kruskal–Wallis test was used to assess homogeneity across non-normally distributed quantitative variables (pre- and postintervention systolic blood pressure, heart rate, respiratory rate, and SpO_2_). This test was also employed to compare pre- and postintervention means for these variables across groups.

Within-group comparisons of these variables before and after the intervention were performed using the Wilcoxon test. For intergroup comparisons of diastolic blood pressure (pre- and postintervention), ANOVA and Tukey's post hoc test were used. The paired *t*-test was applied to assess pre- and postintervention changes in diastolic blood pressure within groups. A significance level of less than 0.05 was adopted for all statistical tests.

### 2.7. Ethical Considerations

This study adhered to the ethical principles outlined in the Helsinki Declaration and received ethical approval from the Arak University of Medical Sciences Ethics Committee (approval code: IR.ARAKMU.REC.1401.032). The study was registered in the Iranian Registry of Clinical Trials (IRCT) under code IRCT20100913004736N27 on June 2, 2022. A formal letter of introduction from Arak University of Medical Sciences was submitted to the Research Deputy of Kermanshah University of Medical Sciences and the relevant authorities at Imam Reza and Taleghani Hospitals in Kermanshah. Informed consent was obtained from all participants after thoroughly explaining the research objectives. Measures were implemented to ensure the confidentiality of participant information and protect patient anonymity.

## 3. Results

**Figure 2:**
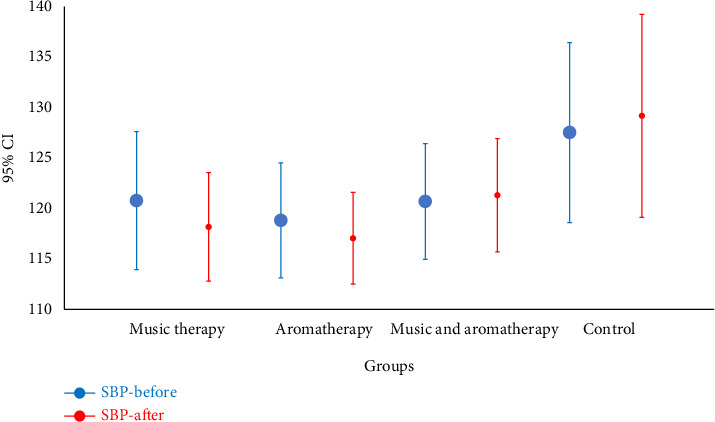
Comparison of mean systolic blood pressure before and after intervention in the study groups. Note: SBP: systolic blood pressure; CI: confidence interval.

**Figure 3:**
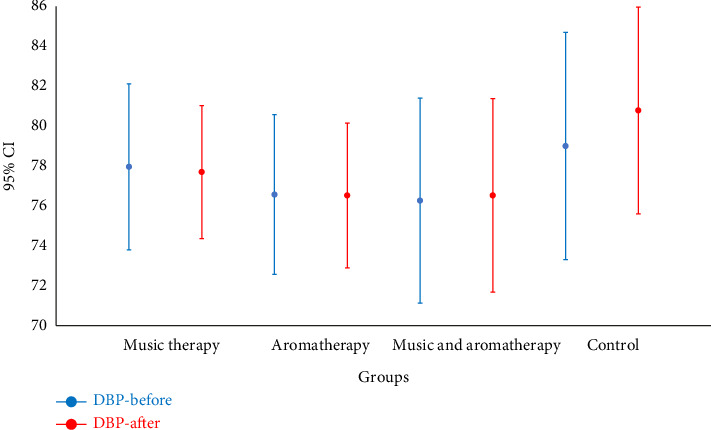
Comparison of mean diastolic blood pressure before and after intervention in the study groups. Note: DBP: diastolic blood pressure; CI: confidence interval.

The mean age of participants was 40.8 ± 2.0 years. The majority were male (*n* = 48, 52.2%), married (*n* = 42, 67.4%), employed (*n* = 72, 73.8%), and had undergone laparotomy surgery (*n* = 62, 67.4%). All four study groups were homogeneous regarding demographic and clinical variables (Table 1). The Kruskal–Wallis test revealed no statistically significant differences between pre-and postintervention systolic and diastolic blood pressure values among the music-based intervention, aromatherapy, combined, and control groups (Figures 2 and 3).

**Figure 4:**
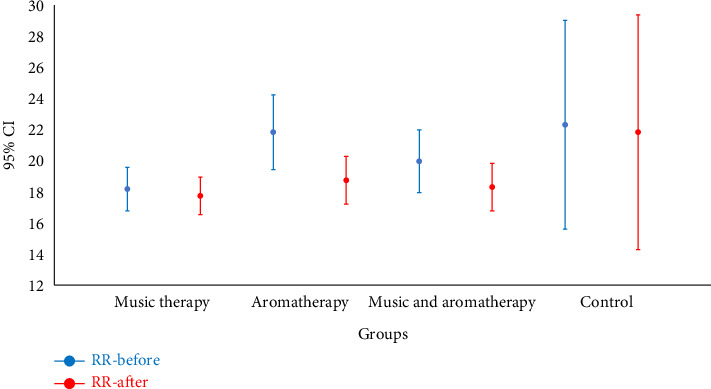
Comparison of the mean respiratory rate before and after intervention in the study groups. Note: RR: respiratory rate; CI: confidence interval.

**Figure 5:**
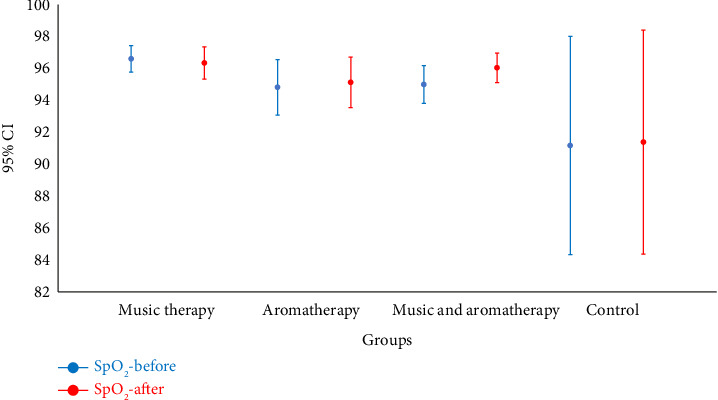
Comparison of mean SpO_2_ before and after intervention in the study groups. Note: SpO_2_: saturation of peripheral oxygen; CI: confidence interval.

In the combined and aromatherapy groups, the postintervention respiratory rate significantly decreased compared to preintervention values (*p*=0.002 and *p* < 0.001, respectively). This difference was not statistically significant in the music therapy and control groups (Figure 4). SpO_2_ levels significantly increased after the intervention in the combined group (*p*=0.020), while no significant change was observed in the other groups (Figure 5).

**Figure 6:**
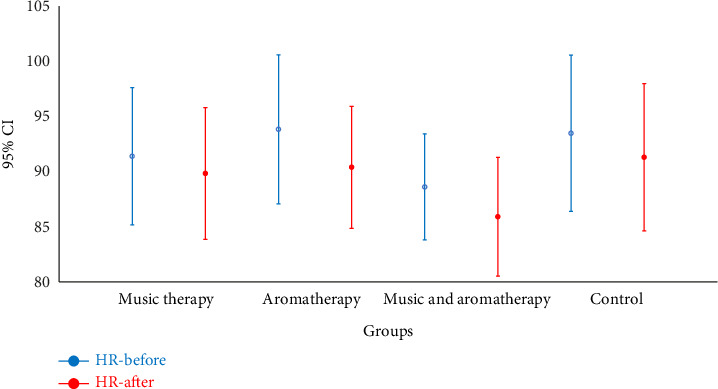
Comparison of mean heart rate before and after intervention in the study groups. Note: HR: heart rate; CI: confidence interval.

In the aromatherapy group, the postintervention pulse rate showed a significant decrease compared to preintervention levels (*p*=0.009). However, this difference was not significant in the other groups (Figure 6) (Table 2).

## 4. Discussion

This randomized controlled trial compared the effects of music-based interventions and aromatherapy on physiological indicators in postoperative ICU patients. The music therapy did not result in statistically significant changes in the measured physiological indicators. However, previous research has reported mixed findings. For instance, a 2019 clinical trial found that music therapy significantly reduced GCS scores, heart rate, and blood pressure in ICU patients [12]. Similarly, a 2019 study involving patients undergoing heart surgery reported significant effects on heart rate, systolic blood pressure, and SpO_2_, but no significant impact on respiratory rate or diastolic blood pressure [14]. In contrast, a 2021 clinical trial noted that music therapy stabilized physiological parameters in ICU patients [11]. Positive effects of music therapy have also been observed in pediatric and adolescent populations. A 2018 clinical trial demonstrated that music therapy stabilized pulse rate, respiratory rate, and systolic and diastolic blood pressure in these patients on the day following surgery [39]. Similarly, a 2022 clinical trial found that music therapy regularized heart rate, respiratory rate, SpO_2_, and blood pressure in patients poststernotomy surgery [10]. A 2023 literature review, synthesizing findings from 18 studies, suggested that music therapy could effectively reduce respiratory rate, heart rate, and blood pressure in ICU patients [3]. In a randomized controlled trial conducted in 2022 involving COVID-19 patients, a single session of music therapy led to a significant improvement in oxygen saturation compared to standard care (97.50 vs. 96.00, *p*=0.026). While heart rate was also measured, the study's primary focus was the increase in O_2_ saturation, underscoring the potential of music therapy as a supportive intervention to improve oxygen levels in clinical settings [5]. The variability in results across studies may be attributed to differences in the type of music, duration and frequency of music exposure, and the characteristics of the patient populations studied. The mechanism through which music therapy exerts its effects may involve a reduction in sympathetic nervous system activity, leading to relaxation and a subsequent decrease in pulse rate, respiratory rate, oxygen consumption, and blood pressure [40].

In this study, inhalation aromatherapy using 25% rosemary essential oil significantly reduced both pulse rate and respiratory rate. These findings are consistent with a 2012 trial that reported a significant reduction in respiratory rate with lavender essential oil aromatherapy, although no significant effects were observed on other physiological indicators [16]. Similarly, a 2019 clinical trial found that aromatherapy with orange and lavender essential oils significantly reduced systolic blood pressure, pulse rate, and respiratory rate in patients undergoing coronary angiography [41]. In contrast, a 2020 clinical trial demonstrated that rose essential oil aromatherapy significantly decreased both systolic and diastolic blood pressure and increased SpO_2_ levels in ICU patients [42]. Another 2022 clinical trial observed that aromatherapy using chamomile and lemon verbena essential oils led to a reduction in heart rate and systolic blood pressure in patients postcoronary angioplasty [26]. However, a 2020 review of 16 articles indicated that while aromatherapy significantly reduced heart rate in patients undergoing heart surgery, it had no significant impact on systolic or diastolic blood pressure [15]. These varied results may be attributed to differences in factors such as the type and concentration of essential oils used, the duration and frequency of the intervention, the number of sessions, and the physiological condition of the patients. Regarding the mechanism of action, evidence suggests that aromatherapy modulates physiological indicators by influencing the autonomic nervous system, increasing parasympathetic activation, lowering cortisol levels, and enhancing melatonin production [43].

In the current study, the combined use of a music therapy and 25% rosemary essential oil aromatherapy had a significant effect on SpO_2_ and respiratory rate, specifically increasing SpO_2_ and decreasing respiratory rate. These results align with a 2017 study, which found significant reductions in heart rate and blood pressure in both the music therapy and aromatherapy groups [4]. Similarly, a 2021 study found that music therapy was more effective than aromatherapy in reducing systolic blood pressure in hypertensive patients, although neither group experienced significant changes in diastolic blood pressure [29]. A 2025 randomized clinical trial investigating music and aromatherapy interventions among patients undergoing endodontic (root canal) treatment reported that although systolic and diastolic blood pressure and heart rate tended to decrease in intervention groups, none of these reductions reached statistical significance compared to the control group [28]. Likewise, a 2020 study comparing music-based intervention, hand massage, and their combination on hospitalized patients' vital signs found that only the combined intervention significantly reduced respiratory rate and heart rate, with no significant effects on blood pressure or SpO_2_ in any group [8]. In contrast, a 2021 study reported that both music therapy and aromatherapy significantly reduced blood pressure in elderly hypertensive patients, with music therapy showing a stronger effect [44]. A 2018 study similarly observed that music therapy significantly decreased systolic and diastolic blood pressure, but not heart rate, in ICU patients. In the same study, the combination of music therapy and aromatherapy led to reductions in diastolic blood pressure and heart rate, but did not affect systolic blood pressure [45]. These diverse findings suggest that the effects of combining music therapy and aromatherapy may vary depending on factors such as music type, duration of music exposure, aroma type and concentration, intervention duration, and the physiological condition of the patients.

### 4.1. Strengths and Limitations of the Study

This study has several key strengths. The use of standardized and calibrated tools ensured the accuracy and reliability of data collection. Measurements of physiological indicators were conducted by the first author, a critical care nursing expert, ensuring measurement validity. Moreover, this study has distinctive strengths. First, it employed a novel intervention that combined music with 25% rosemary essence aromatherapy, an approach not previously explored. Second, the randomized block design helped minimize potential biases and enhanced internal validity, allowing for more robust between-group comparisons. Notably, the study experienced no sample attrition, which reduced the risk of bias and enhanced the reliability of the results. Having complete data from all participants enabled a comprehensive analysis. In addition, blinding the statistical consultant during data analysis further enhanced objectivity and reduced bias. Lastly, allowing participants to select their preferred music type added an element of personalization, potentially increasing both intervention effectiveness and participant engagement.

Despite its strengths, this study also has limitations. First, the scarcity of comparable studies on surgical ICU patients complicates cross-population comparisons of findings. Second, the relatively brief 30-min intervention duration limits a comprehensive evaluation of long-term effects. Third, assessing physiological indicators immediately after the intervention, without using a sequential measurement approach, limits the understanding of the intervention's prolonged impact. Fourth, restricting patients to a predefined list of six music options, without fully considering individual preferences, may have influenced their subjective responses. Finally, the study was conducted in only two healthcare centers, which limits the generalizability of the findings to broader populations.

## 5. Conclusion

This study compared the effects of music therapy and rosemary essence aromatherapy on physiological indicators in surgical ICU patients. The results demonstrated significant improvements in certain physiological indicators following the interventions, offering important implications for both patients and ICU nurses. Implementing these interventions in clinical settings could help stabilize and improve patients' physiological indicators, potentially leading to better outcomes. Specifically, significant reductions in respiratory rate were observed in both the combined intervention and aromatherapy groups when compared to preintervention values. In addition, the combined group exhibited significant increases in arterial oxygen saturation, while the aromatherapy group experienced significant decreases in pulse rate postintervention. Given the positive impact of both aromatherapy and music-based interventions on physiological indicators, it is recommended that these simple, safe, and noninvasive interventions be incorporated into the care of surgical ICU patients.

Future research should focus on extending the duration of interventions and assessing their effects at multiple time points to gain a more comprehensive understanding of their long-term impact. It is also essential to explore the feasibility and generalizability of these interventions in ICUs and other hospital departments. Further investigations into their applicability and potential benefits across diverse patient populations, including both surgical and nonsurgical patients, are necessary. In addition, future studies should explore the influence of demographic variables such as age, gender, and diagnosis to provide more specific insights into the effectiveness of these interventions for targeted patient populations. Evaluating the cost-effectiveness and practicality of implementing these interventions on a larger scale may significantly influence their acceptance and integration into healthcare facilities.

## Figures and Tables

**Table 1:** Demographic characteristics of samples in music-based intervention, aromatherapy, combination of music and aromatherapy, and control groups.

Variables		Groups				*p* value
		Music-based intervention	Aromatherapy	Combined	Control	
Age, mean ± SD^¥^		37.6 ± 15.3	40.6 ± 13.8	45.1 ± 13.6	40.0 ± 15.1	0.362

Gender, *n* (%)	Male	12 (52.2)	14 (60.9)	12 (52.2)	9 (39.1)	0.707
	Female	11 (47.8)	9 (39.1)	11 (47.8)	13 (56.5)	

Education	Primary	9 (39.1)	5 (21.7)	8 (34.8)	8 (34.8)	0.680
	Diploma	6 (26.1)	8 (34.8)	7 (30.4)	10 (43.5)	
	University	8 (34.8)	10 (43.5)	8 (34.8)	5 (21.7)	

Marital status	Single	8 (34.8)	12 (52.2)	4 (17.4)	6 (26.1)	0.074
	Married	15 (65.2)	11 (47.8)	19 (82.6)	17 (73.9)	

Occupation	Employed	15 (65.2)	16 (69.6)	21 (91.3)	20 (87.0)	0.084
	Unemployed	8 (34.8)	7 (30.4)	2 (8.7)	3 (13.0)	

Settlement	Urban	20 (87.0)	17 (73.9)	19 (82.6)	16 (69.6)	0.465
	Rural	3 (13.0)	6 (26.1)	4 (17.4)	7 (30.4)	

Surgery history	Yes	11 (47.8)	10 (43.5)	10 (43.5)	13 (56.5)	0.790
	No	12 (52.2)	13 (56.5)	13 (56.5)	10 (43.5)	

Surgery type	Laparotomy	18 (78.3)	15 (65.2)	14 (60.9)	15 (65.2)	0.619
	Thoracotomy	5 (21.7)	8 (34.8)	9 (39.1)	8 (34.8)	

Receiving analgesic	Yes	7 (30.4)	10 (43.5)	4 (17.4)	7 (30.4)	0.296
	No	16 (69.6)	13 (56.5)	19 (82.6)	16 (69.6)	

^¥^Standard deviation.

**Table 2:** Comparison of physiological indices in music-based intervention, aromatherapy, combination of music and aromatherapy, and control groups.

Variables	Times	Groups				*p* value
		Music therapy	Aromatherapy	Combined	Control	
		Median ± IQR^€^	Median ± IQR	Median ± IQR	Median ± IQR	
Pulse rate	Before	91.4 ± 14.4	93.8 ± 15.6	88.6 ± 11.1	93.5 ± 16.4	0.722
	After	89.8 ± 13.8	90.4 ± 12.8	85.9 ± 12.4	91.3 ± 15.4	0.684
	*p* value^¥^	0.348	0.009	0.055	0.794	—

Respiration rate	Before	18.8 ± 3.2	21.8 ± 5.5	19.9 ± 4.6	22.3 ± 15.5	0.212
	After	17.7 ± 2.8	18.7 ± 3.5	18.3 ± 3.5	21.8 ± 17.4	0.682
	*p* value	0.275	< 0.001	0.002	0.199	—

Systolic blood pressure	Before	120.8 ± 15.8	118.8 ± 13.1	120.7 ± 13.2	127.5 ± 20.6	0.408
	After	118.2 ± 12.4	117.0 ± 10.5	121.3 ± 12.9	129.2 ± 23.2	0.167
	*p* value	0.094	0.442	0.664	0.702	—

Diastolic blood pressure	Before	77.9 ± 9.6	76.6 ± 9.6	76.3 ± 11.9	79.0 ± 13.2	0.824
	After	77.7 ± 7.7	76.5 ± 8.4	76.5 ± 11.2	80.8 ± 11.9	0.428
	*p* value	0.853	0.866	0.866	0.403	—

PaO_2_	Before	96.6 ± 1.9	94.8 ± 4.0	95.0 ± 2.7	91.2 ± 15.8	0.021
	After	97.3 ± 2.3	95.1 ± 3.6	96.0 ± 2.1	91.4 ± 16.2	0.127
	*p* value	0.590	0.424	0.020	0.468	—

^¥^Based on the Wilcoxon signed-rank test.

^€^Interquartile range.

## Data Availability

Data are available upon reasonable request.

## Supporting Information

CONSORT 2010 checklist: The CONSORT 2010 checklist is a supporting file that ensures comprehensive reporting of a randomized controlled trial according to the CONSORT 2010 statement.

## References

[1] Mollaoğlu M. C., Karabulut O., Boy Y., Mollaoğlu M., Karadayı K. (2022). Environmental Stressors Perceived by Patients in the Surgical Intensive Care Unit. *Turkish Journal of Intensive Care*.

[2] Sarıtaş S. Ç., Araç B. (2016). The Effect of Music Therapy on the Vital Signs of Patients in a Surgical Intensive Care Unit. *International Journal of Medical Investigation*.

[3] Lorek M., Bąk D., Kwiecień-Jaguś K., Mędrzycka-Dąbrowska W. (2023). *The Effect of Music as a Non-Pharmacological Intervention on the Physiological, Psychological, and Social Response of Patients in an Intensive Care Unit*.

[4] Lee C.-H., Lai C.-L., Sung Y.-H., Lai M. Y., Lin C.-Y., Lin L.-Y. (2017). Comparing Effects Between Music Intervention and Aromatherapy on Anxiety of Patients Undergoing Mechanical Ventilation in the Intensive Care Unit: A Randomized Controlled Trial. *Quality of Life Research*.

[5] Giordano F., Losurdo A., Quaranta V. N. (2022). Effect of Single Session Receptive Music Therapy on Anxiety and Vital Parameters in Hospitalized COVID-19 Patients: A Randomized Controlled Trial. *Scientific Reports*.

[6] Golino A. J., Leone R., Gollenberg A. (2019). Impact of an Active Music Therapy Intervention on Intensive Care Patients. *American Journal of Critical Care*.

[7] Stea S., Beraudi A., De Pasquale D. (2014). Essential Oils for Complementary Treatment of Surgical Patients: State of the Art. *Evidence-based Complementary and Alternative Medicine*.

[8] Elay G., Ozkaya M. (2020). The Effect of Music and Massage on the Pain Scales and Vital Signs of ICU Patients With Hemodialysis Catheter. *European Journal of Therapeutics*.

[9] Goli R., Arad M., Mam-Qaderi M., Parizad N. (2022). Comparing the Effects of Geranium Aromatherapy and Music Therapy on the Anxiety Level of Patients Undergoing Inguinal Hernia Surgery: A Clinical Trial. *Explore*.

[10] Ganesan P., Manjini K. J., Bathala Vedagiri S. C. (2022). Effect of Music on Pain, Anxiety and Physiological Parameters Among Postoperative Sternotomy Patients: A Randomized Controlled Trial. *Journal of Caring Sciences*.

[11] Chahal J. K., Sharma P., Rawat H., Rawat H. (2021). Effect of Music Therapy on ICU Induced Anxiety and Physiological Parameters Among ICU Patients: an Experimental Study in a Tertiary Care Hospital of India. *Clinical Epidemiology and Global Health*.

[12] Raisinghani N., Jawaharani A., Acharya S., Kumar S., Gadegone A. (2019). The Effect of Music Therapy in Critically Ill Patients Admitted to the Intensive Care Unit of a Tertiary Care Center. *Journal of Datta Meghe Institute of Medical Sciences University*.

[13] Kühlmann A. Y. R., Etnel J. R., Roos-Hesselink J. W., Jeekel J., Bogers A. J., Takkenberg J. J. (2016). Systematic Review and Meta-Analysis of Music Interventions in Hypertension Treatment: A Quest for Answers. *BMC Cardiovascular Disorders*.

[14] Mirbagher Ajorpaz N., Mohammadi A., Najaran H., Khazaei S. (2019). Effect of Music on Postoperative Physiological Parameters in Patients Under Open Heart Surgery. *Journal of Research and Health*.

[15] Abdelhakim A. M., Hussein A. S., Doheim M. F., Sayed A. K. (2020). The Effect of Inhalation Aromatherapy in Patients Undergoing Cardiac Surgery: A Systematic Review and Meta-Analysis of Randomized Controlled Trials. *Complementary Therapies in Medicine*.

[16] Büyükbayram Z., Zengin Aydin L., Araç E. (2021). The Effect of Aromatherapy Application on the Vital Signs of Intensive Care Patients. *International Journal of Traditional and Complementary Medicine Research*.

[17] Estores I. M., Frye J. (2015). Healing Environments: Integrative Medicine and Palliative Care in Acute Care Settings. *Critical Care Nursing Clinics of North America*.

[18] Rashidi Fakari F., Tabatabaeichehr M., Kamali H., Rashidi Fakari F., Naseri M. (2015). Effect of Inhalation of Aroma of Geranium Essence on Anxiety and Physiological Parameters During First Stage of Labor in Nulliparous Women: A Randomized Clinical Trial. *Journal of Caring Sciences*.

[19] Gonçalves G. A., Corrêa R. C. G., Barros L. (2019). Effects of In Vitro Gastrointestinal Digestion and Colonic Fermentation on a Rosemary (*Rosmarinus officinalis* L) Extract Rich in Rosmarinic Acid. *Food Chemistry*.

[20] Cedillo-Portillo J. J., Villastrigo-López W. Y., Castañeda-Facio A. O., Esparza-González S. C., Múzquiz-Ramos E. M., Sáenz-Galindo A. (2024). *Salvia rosmarinus* Spenn. Main Applications and Ultrasonic Extraction of Secondary Metabolites: A General Review. *Revista Mexicana de Ingeniería Biomédica*.

[21] US Food and Drug Administration (2022). *Substances Added to Food (Formerly EAFUS)*.

[22] Azizi S., Mohamadi N., Sharififar F., Dehghannoudeh G., Jahanbakhsh F., Dabaghzadeh F. (2022). Rosemary as an Adjunctive Treatment in Patients With Major Depressive Disorder: A Randomized, Double-Blind, Placebo-Controlled Trial. *Complementary Therapies in Clinical Practice*.

[23] Mank-Halati M. S., Rezaei M., Farzaei M. H., Khatony A. (2024). Comparing the Effects of Rosemary Aromatherapy and Music Therapy on Anxiety Levels in Patients Undergoing General Surgery: A Randomized Controlled Clinical Trial. *Explore*.

[24] Rahbardar M. G., Hosseinzadeh H. (2020). Therapeutic Effects of Rosemary (*Rosmarinus officinalis* L.) and Its Active Constituents on Nervous System Disorders. *Iranian journal of basic medical sciences.*.

[25] Isabel A. A., Cristina R. I., Laurindo P. (2016). Effects of Music Therapy in Intensive Care Patients. *International Journal of Nursing*.

[26] Asadi Mobarakeh M., Ziaeirad M. (2022). Comparing the Effect of Aromatherapy With Geranium and Lemon Essential Oil on Situational Anxiety and Physiological Indices of Patients After Coronary Angioplasty. *Complementary Medicine Journal*.

[27] Ghasemi S., Babatabar D., Ebadi A. (2017). Investigating of the Effect of Aromatherapy With Rose on Physiologic Parameters and Mechanical Ventilation Weaning Time in Patients Undergoing open-heart Surgery. *Journal of the Iranian Society of Anesthesiology and Critical Care*.

[28] Mousavi S. A., Nasiri F., Kashani M. S., Ghazalgoo A., Iranmanesh P., Shahbazi S. (2025). Effects of Music and Aromatherapy on Blood Pressure and Heart Rate Among Endodontic Patients: A Randomized Clinical Trial. *Clinical and Experimental Dental Research*.

[29] Ubaidillah Z., Rahayu H., Ruhyanudin F. (2022). Comparing Effects Between Music Intervention and Aromatherapy on Blood Pressure Among Hypertensive Patients: A Feasibility Study Conducted in Indonesia. *Malaysian Journal of Medicine and Health Sciences*.

[30] Liu Y., Tang Q., Zhao X. (2021). Neural Activation of Different Music Styles During Emotion-Evoking. *Psychology of Music*.

[31] Vuust P., Heggli O. A., Friston K. J., Kringelbach M. L. (2022). Music in the Brain. *Nature Reviews Neuroscience*.

[32] Pant U., Frishkopf M., Park T., Norris C. M., Papathanassoglou E. (2022). A Neurobiological Framework for the Therapeutic Potential of Music and Sound Interventions for Post-Traumatic Stress Symptoms in Critical Illness Survivors. *International Journal of Environmental Research and Public Health*.

[33] Giordano F., Mitrotti A., Losurdo A. (2023). Effect of Music Therapy Intervention on Anxiety and Pain During Percutaneous Renal Biopsy: A Randomized Controlled Trial. *Clinical Kidney Journal*.

[34] Giordano F., Giglio M., Sorrentino I. (2023). Effect of Preoperative Music Therapy Versus Intravenous Midazolam on Anxiety, Sedation and Stress in Stomatology Surgery: A Randomized Controlled Study. *Journal of Clinical Medicine*.

[35] Baker T. B., Smith S. S., Bolt D. M. (2017). Implementing Clinical Research Using Factorial Designs: A Primer. *Behavior Therapy*.

[36] Andrade C. (2024). Understanding Factorial Designs, Main Effects, and Interaction Effects: Simply Explained With a Worked Example. *Indian Journal of Psychological Medicine*.

[37] Sharma M.-P., Cetera B. (2020). Thyroid Disease and Surgery. *Anaesthesia and Intensive Care Medicine*.

[38] Sundaram V., Selvaganesan P., Deo S., Karnib M. (2022). The Importance of Randomization in Clinical Research. *Indian Journal of Thoracic and Cardiovascular Surgery*.

[39] Karakul A., Bolışık Z. B. (2018). The Effect of Music Listened to During the Recovery Period After Day Surgery on the Anxiety State and Vital Signs of Children and Adolescents. *The Journal of Pediatric Research*.

[40] Parada-Cabaleiro E., Batliner A., Schuller B. W. (2021). The Effect of Music in Anxiety Reduction: A Psychological and Physiological Assessment. *Psychology of Music*.

[41] Tahmasebi H., Poorkhiz A., Abdi Joubari H. (2019). Comparing the Aromatherapeutic Effects of Orange and Lavender Essential Oils on Anxiety and Physiological Indicators in Patients Undergoing Coronary Angiography: A Clinical Trial Study. *Medical-Surgical Nursing Journal*.

[42] Zare N., Shahabinejad M., Sadeghi T. (2020). The Effect of Aromatherapy by Rose Essence on Anxiety and Physiological Indices of Conscious Patients Admitted at Intensive Care Units. *Hormozgan Medical Journal*.

[43] Bahrami T., Rejeh N., Heravi‐Karimooi M., Vaismoradi M., Tadrisi S. D., Sieloff C. (2017). Effect of Aromatherapy Massage on Anxiety, Depression, and Physiologic Parameters in Older Patients With the Acute Coronary Syndrome: A Randomized Clinical Trial. *International Journal of Nursing Practice*.

[44] Kurniasih D. E., Erwanto R. (2021). The Effectiveness Differences of Cananga Aromatherapy and Java Langgam Music on Blood Pressure of the Elderly With Hypertension in Bpstw Yogyakarta. *Jurnal Keperawatan Respati Yogyakarta*.

[45] Kumala F., Fatmasari D., Lestari K. P., Hadisaputro S. (2018). Music and Aromatherapy: A Good Combination for Reducing Anxiety and Stabilizing Non-Invasive Hemodynamic Status in Patients in the Intensive Care Unit. *Belitung Nursing Journal*.

[46] Bolandi Z., Khatony A. R., Rezaei M., Mahbobeh Sajadi M. (2022). *Comparison of the Effect of Music Therapy and Aromatherapy on the Level of Anxiety and Physiological Index of Post-Surgery Patients Hospitalized in the Intensive Care Units*.

